# Enhanced Expression but Decreased Specific Activity of Matrix Metalloproteinase 10 (MMP-10) in Comparison with Matrix Metalloproteinase 3 (MMP-3) in Human Urinary Bladder Carcinoma

**DOI:** 10.3390/jcm10163683

**Published:** 2021-08-19

**Authors:** Jacek Kudelski, Grzegorz Młynarczyk, Monika Gudowska-Sawczuk, Barbara Mroczko, Barbara Darewicz, Marta Bruczko-Goralewska, Krzysztof Sobolewski, Lech Romanowicz

**Affiliations:** 1 Department of Urology, Medical University of Bialystok, M. Skłodowskiej-Curie 24A St., 15-276 Białystok, Poland; mlynarz36@yahoo.pl (G.M.); barbara.darewicz@umb.edu.pl (B.D.); 2 Department of Medical Biochemistry, Medical University of Bialystok, Adama Mickiewicza 2C St., 15-089 Białystok, Poland; marta.bruczko-goralewska@umb.edu.pl (M.B.-G.); zdbioch@umb.edu.pl (K.S.); lech.romanowicz@umb.edu.pl (L.R.); 3 Department of Biochemical Diagnostics, Medical University of Bialystok, Waszyngtona 15A St., 15-269 Bialystok, Poland; monika.gudowska-sawczuk@umb.edu.pl (M.G.-S.); mroczko@umb.edu.pl (B.M.); 4 Department of Neurodegeneration Diagnostics, Medical University of Bialystok, Waszyngtona 15A St., 15-269 Bialystok, Poland

**Keywords:** urinary bladder carcinoma, MMP-3, MMP-10, cancer, biomarker

## Abstract

Human urinary bladder cancer is a huge worldwide oncological problem causing many deaths every year. The degradation of extracellular matrix (ECM) induced by molecules such as matrix metalloproteinases (MMPs) is one of the main factors influencing the process of metastasis origination. The MMP expression is tied to tumor aggressiveness, stage, and patient prognosis. The cleavage of constituent proteins is initiated and prolonged by matrix metalloproteinases, such as MMP-3 and MMP-10. The aim of this study was to evaluate the expression and activity of both MMPs in human urinary bladder cancer occurring at various stages of the disease. Tissue samples from patients with urinary bladder cancer were analyzed. Samples were collected from patients with a low- and high-grade cancer. Control tissue was collected from the site opposite to the tumor. DNA content, MMPs content, and activity of MMP-3 and MMP-10 were measured using ELISA and Western blot techniques. MMP-3 and MMP-10 occur in high molecular complexes in human urinary bladder in healthy and cancerous tissues. Particularly, in high-grade tumors, the content of MMP-10 prevails over MMP-3. The actual and specific activities vary in both grades of urinary bladder cancer; however, the highest activity for MMP-3 and MMP-10 was found in low-grade tissues. In conclusion, MMP-10 had a higher content, but a lower activity in all investigated tissues compared to MMP-3. Generally, obtained results demonstrated a contrary participation of MMP-3 and MMP-10 in ECM remodeling what may be crucial in the pathogenesis of human urinary bladder carcinoma.

## 1. Introduction

The urinary bladder has a well-developed muscular tissue, usually arranged in bundles accompanied by elastic fibres [1,2,3,4,5]. The bladder itself consists of four layers [1,2,4]. The epithelium in contact with the urine is called transitional epithelium. Most bladder cancers originate from the transitional epithelial cells. Lamina propria is a layer of blood vessels and connective tissue. The true muscular bladder layer referred to as muscularis propria or detrusor muscle is the third layer of the bladder. The outermost fourth layer is composed of fat, fibrous tissue, and blood vessels [1,2].

Bladder cancer is very common malignancy in the world, and it is estimated that about 550,000 new cases are diagnosed yearly [6,7,8]. The type of cancer is described by its grade under microscope. If cancerous tissues are similar to healthy ones, they are called well-differentiated or low-grade (LG) cells. Cancer cells that differ morphologically from normal cells are named poorly-differentiated or high-grade (HG) cells. Low-grade tumors involve a much lower risk of progression than high-grade tumors [2].

The conducted research studies showed that some matrix metalloproteinases are associated with metastases in various cancers, i.e., bladder cancer [9,10]. Matrix metalloproteinases are an enzyme family with a specific feature that is a multi-domain structure consisting of the following elements: pro-domain, catalytic domain, hinge region, and hemopexin domain. Their main function is to degrade the extracellular matrix components. The MMPs activity is regulated at the different stages such as transcription, secretion from cells, activation, and by tissue inhibitors of metalloproteinases (TIMPs). The matrix degradation enables the cells to migrate. This process is called extracellular matrix remodeling. The activity of MMPs is significantly increased in some pathological conditions, e.g., they play a significant role in tumorgenesis [11,12,13,14,15,16,17]. However, the expression of MMP-10 likely does not show any relationship with the progression of urinary bladder cancer [18,19].

Some matrix metalloproteinases are also known as stromelysines. Stromelysines are formed by the following three enzymes: MMP-3 (stromelysin 1), MMP-10 (stromelysin 2), and MMP-11 (stromelysin 3). Among them, stromelysin-1 demonstrates the highest proteolytic activity as well as the largest ability of activation of other inactive metalloproteinases-zymogens [20,21,22].

Stromelysin-1 degrades collagen types II, III, IV, IX, X, and XI also aggrecan, elastin, fibronectin, gelatin, laminin, proteoglycans, MMP-7, -8, and -13. In addition, it has a higher proteolytic potential than stromelysin-2 [20,21,22,23,24]. Stromelysin-2 degrades collagen types: III, IV, and V as well as aggrecan, elastin, fibronectin, gelatin, laminin, MMP-1, and -8. The gene of both MMPs is localized on chromosome 11 [25,26,27].

Various research studies on the content and activity of MMPs in urinary bladder cancer frequently brought different results. Such differences could result from the methodology and the tissue material. Dominating outcomes were assayed in blood and urine of patients with cancer. These results seem to be not entirely adequate for an association of MMPs with the carcinogenesis process. Considering this observation, we evaluated the content, expression, and activity of two stromelysines: MMP-3 and MMP-10 in healthy and cancerous tissues.

## 2. Materials and Methods

The research was approved by the Bioethical Committee of the Medical University of Bialystok.

### 2.1. Tissue Material

The research related to urothelial cancer, and comprised two stages of morphological malignancy. The material was obtained in the course of surgical procedures at the Department of Urology of the Medical University of Bialystok. The study was conducted on twenty patients who underwent radical cystectomy or transurethral resection of the bladder tumor with the histopathological diagnosis of urothelial cancer. The patients were enrolled to our study consecutively, basing on histopathological outcome. We excluded patients who received neoadjuvant chemotherapy, intravesical treatment, or pelvic radiotherapy. All patients treated with transurethral resection of bladder tumor (TURBT) were enrolled to the study only if it was their first urological, invasive treatment. After transurethral resection of the tumor, only a part of the whole material was taken to the study. Most of it was send for histopathological examination. There was no cauterization artefact noticed by pathologist and the diagnosis was stated with high accuracy. Considering this fact, there was very low potential of cauterization artefact considering biochemical methods. All patients who underwent endoscopic treatment, they were treated with monopolar resection. The age span was 47–91 years and the average age was 70.3 years.

The urinary bladder excision was performed using the classical, open approach, and a tissue sample was taken from the macroscopically visible tumor. The patients consisted of two groups: 10 patients diagnosed with a low-grade cancer (treated with TURBT procedure) and 10 patients diagnosed with a high-grade cancer (treated with radical cystectomy). The control tissue was collected following the radical open cystectomy from the side opposite to the tumor. It was not possible to collect a healthy tissue during the transurethral resection of the bladder tumor. The following abbreviations are used hereafter: LG—low-grade cancer, HG—high-grade cancer, and control—control tissue.

### 2.2. DNA Content

The content of DNA in all investigated tissues was determined with the method described by Burton [28]. The principle of the method is specific colorimetric reaction of diphenylamine with deoxyribose in acidic solution in boiling water bath.

### 2.3. Content of MMPs (Elabscience Biotechnology, Human TMP-3 ELISA Kit)

The content of MMP-3 in the investigated samples was determined with the quantitative assay, Quantikine ELISA Human Total MMP-3 Kit (provided by R&D systems, Minneapolis, MN, USA) and MMP-10 content with Human Stromelysin-2 ELISA Kit (EIAab; Wuhan, China) as per manufacturer’s instructions.

### 2.4. Western Blot of MMPs (Abcam USA, Cat No. ab 85926 Policlonal Antibody)

The samples (20 μg of protein) of tissue extracts of normal urinary bladder and both kinds of cancers were electrophoresed on 10% SDS-polyacrylamide gel according to the method of Laemmli [29], and blotted to nitrocellulose membranes (Sigma-Aldrich; Saint Louis, MO, USA) at 100 mA for 1 h. The membranes were blocked with 5% (*w*/*v*) non-fat powdered milk in TBS-T solution (20 mM Tris/HCl buffer, pH 7.4, 150 mM NaCl, 0.05% (*v*/*v*) Tween 20) for 1 h. They were then incubated with monoclonal antibody directed against human MMP-3 (catalogue number MAB905; R&D Systems, Minneapolis, MN, USA) or monoclonal antibody directed against human MMP-10 (catalogue number MAB910; R&D Systems; Minneapolis, MN, USA) in TBS-T containing 1% bovine serum albumin (*w*/*v*), overnight at 4 °C. After several washes in TBS-T buffer, bound antibodies were detected using alkaline phosphatase-conjugated with respective secondary antibody in the same solution, for 1 h, at room temperature with gentle mixing and then BCIP/NBT reagent (catalogue number B1911; Sigma-Alrdrich; Saint Louis, MO, USA). The molecular mass of MMPs was estimated using pre-stained molecular mass markers (BioRad, Hercules, CA, USA). Representative blots were shown.

### 2.5. MMPs Activity

The assessment of MMP-3 and MMP-10 actual specific activity was performed in a black 96-flat-bottom-well microplate (Greiner Bio-One, Rainbach im Mühlkreis, Austria) which was pre-coated with a respective specific MMP antibody (the same antibodies that were used in the Western blot analysis) [30]. One hundred microliters of appropriate sample were added to each well in order to immobilize the metalloproteinase. The microplate was incubated overnight at 4 °C. All other proteins were washed out with TBS-T buffer (50 mM of Tris/HCl pH 7.4, 0.9% NaCl, 0.05% Tween 20). The MMP activity was assessed in 100 μL of 50 mM Tris/HCl buffer pH 7.5 containing 10 mM of CaCl_2_, 150 mM of NaCl, and 0.025% Brij 35 [7] with MCA-Pro-Leu-Ala-Cys(p-OMeBz)-Trp-Ala-Arg(Dpa)-H2 (Merck, Germany) as a fluorogenic substrate (4 μM of final concentration). The microplate was incubated at 37 °C for 60 min with gentle shaking. The reaction was stopped by the addition of 25 μL of 100 mM of EDTANa_2_. The degradation of the fluorogenic substrate was assessed with a multimode microplate reader (Tecan Infinite^®^ 200 PRO, Männedorf, Switzerland) with the excitation and emission wavelengths of 325 and 393 nm, respectively. The quantity of degraded substrate was calculated from the calibration curve obtained in the same conditions with 7-amino-4-methylcoumarin (Sigma–Aldrich, Saint Louis, MO, USA) as a standard.

The actual activity of investigated metalloproteinase was calculated by the dividing of the quantity of degraded fluorogenic substrate by separated enzyme by total protein content in tissue extract. The specific activity of investigated metalloproteinase was calculated by the dividing of the quantity of degraded fluorogenic substrate by separated enzyme by the enzyme protein content in tissue extract.

The MMP specific activity was stated in katals per kg of protein.

### 2.6. Protein Determination

The protein concentration was assessed with the method described by Bradford [31]. Determination is based on specific colorimetric reaction of Coomassie Brilliant Blue G-250 with peptides and proteins with molecular mass higher than 3000 Da.

### 2.7. Statistical Analysis

The results were presented as mean values ± standard deviations (SD). The matrix metalloproteinases content was given in nmol/g of fresh tissue. Their activity was given in microkat/kg of protein. The results were statistically analyzed using the Student’s *t*-test with the statistical significance of *p* ˂ 0.05.

## 3. Results

(1) DNA content

**Figure 1:**
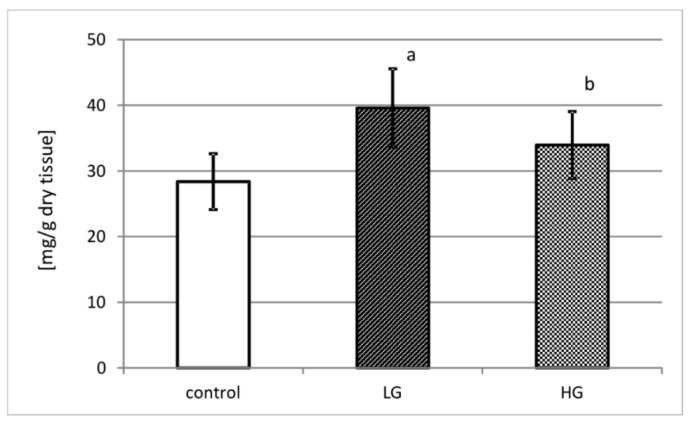
DNA content in control tissue and low-grade (LG) and high-grade (HG) urinary bladder cancer. a—*p* < 0.001 low-grade cancer vs. urinary bladder control; b—*p* < 0.05 high-grade vs. low-grade urinary bladder cancer.

Figure 1 demonstrates DNA content in control urinary bladder tissue and in low- and high-grade urinary bladder cancer in miligrams recalculated per gram of dry tissue. Control urinary bladder consists of approximately 28 mg of DNA. Both grades of cancer present higher content of deoxyribonucleic acid than control tissue. In low-grade cancer, an increase of more than 30% of DNA content comparing to control (*p* < 0.001) was observed. High-grade cancer tissue presented 17% increase of DNA content in comparison with control tissue (*p* < 0.05).

(2) MMP-3 and MMP-10 content

**Figure 2:**
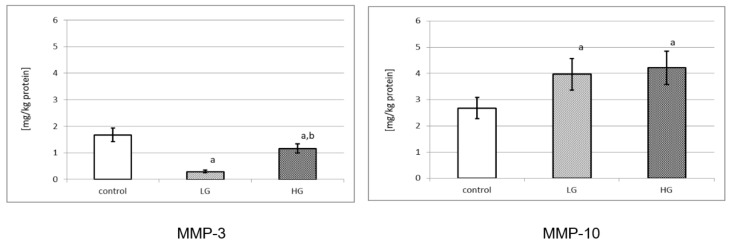
Content of MMP-3 and MMP-10 in control tissue and low-grade (LG) and high-grade (HG) urinary bladder cancer. a—*p* < 0.001 cancer vs. urinary bladder control; b—*p* < 0.001 high-grade vs. low-grade urinary bladder cancer.

Figure 2 demonstrates the amount of MMPs in control and cancer tissues. Both MMPs were expressed in normal urinary bladder and in bladder cancer. MMP-3 was present in a control tissue extract in the amount of 1.675 mg/kg of protein. Control tissue had a significantly higher amount of that enzyme in comparison to low-grade and high-grade urinary bladder cancer (*p* < 0.001 for both). Moreover, high-grade cancer had significant increase in the amount of MMP-3 in comparison to low-grade cancer (*p* < 0.001). The content of MMP-10 was significantly lower in control tissue in comparison to low-grade and high-grade cancer tissues (*p* < 0.001 for both). In addition, the content of MMP-10 in the control tissue, low-grade cancer and high-grade cancer was higher in comparison to MMP-3.

(3) Western blot analysis of investigated stromelysines

The Western blot analysis by electrophoresis was performed in reducing and non-reducing conditions with the same protein amount in the samples.

### 3.1. Expression of MMP-3 in Human Urinary Bladder

**Figure 3:**
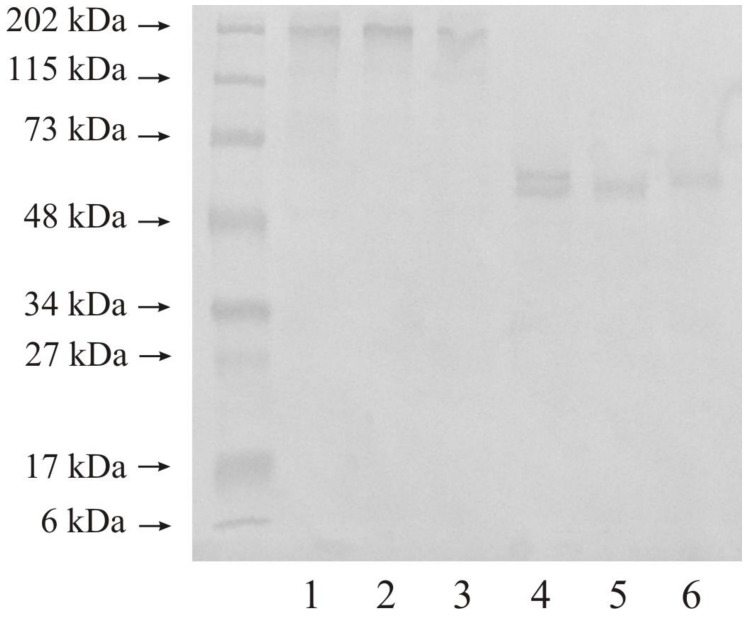
Western immunoblot of MMP-3 in control tissue and low-grade (LG) and high-grade (HG) urinary bladder cancer. Twenty micrograms of protein: lane 1—control urinary bladder, 2—low-grade bladder cancer, 3—high-grade bladder cancer; twenty microgram protein: lane 4—control urinary bladder, 5—low-grade bladder cancer, 6—high-grade bladder cancer.

Figure 3 shows the expression of MMP-3 in normal urinary bladder as well as in tissues changed by carcinogenic processes. We used 20 micrograms of protein on lanes 1–3 and 20 micrograms of protein on lanes 4–6 of the same samples. Normal urinary bladder demonstrated only one band with a molecular mass of 202 kDa (lane 1). Low-grade urinary bladder cancer also had one band with a similar molecular mass of 202 kDa (lane 2). The tissue of high-grade urinary bladder cancer showed the same results as control and low-grade tissues (lane 3). The reduction conditions revealed only one visible band of 50 kDa. All three kinds of samples demonstrated the same result kDa (lane 4–6).

### 3.2. Expression of MMP-10 in Human Urinary Bladder

**Figure 4:**
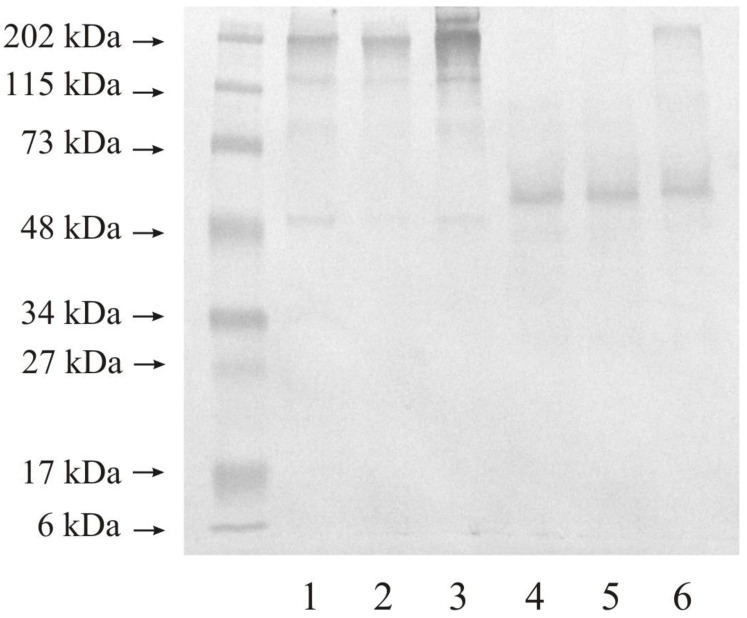
Western immunoblot of MMP-10 in control tissue and low-grade (LG) and high-grade (HG) urinary bladder cancer. Twenty micrograms of protein: lane 1—control urinary bladder, 2—low-grade bladder cancer, 3—high-grade bladder cancer; twenty micrograms of protein: lane 4—control urinary bladder, 5—low-grade bladder cancer, 6—high-grade bladder cancer.

Figure 4 shows the results MMP-10 expression in healthy urinary bladder as well as in tissues changed by carcinogenic processes. We used 20 micrograms of protein on lanes 1–3, and 20 micrograms of protein on lanes 4–6 of the same samples. Control sample demonstrated at least three bands with the following molecular mass: narrow band of 48 and 115 kDa, and wide band of 202 kDa (lane 1). Low-grade urinary bladder cancer (lane 2) and high-grade urinary bladder cancer (lane 3) tissues showed results closed to the control tissue. The reduction of disulfide linkage resulted in the reduction of the number of present bands. The used anti-MMP- antibody makes visible proteins with the molecular mass of about 50 kDa in all samples (lane 4–6) and with a higher molecular mass of about 202 kDa in high-grade cancer (lane 6).

(4) Actual activity of MMP-3 and MMP-10

The actual activity of collagenases was determined with the use of the fluorimetric method with oligopeptide applied as a substrate. Enzymes were separated on a microplate pre-coated with specific for the collagenase antibodies to be determined. The actual activity was expressed in katals per kg of total protein content in the tissue extract.

**Figure 5:**
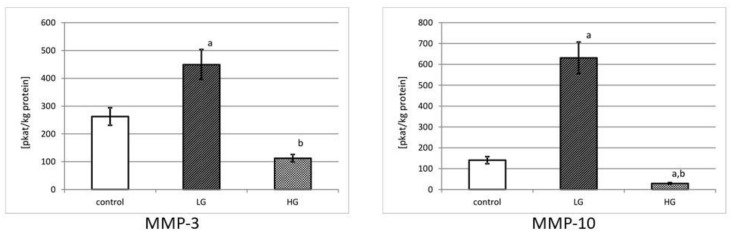
MMP-3 and MMP-10 actual activity in control tissue and low-grade (LG) and high-grade (HG) urinary bladder cancer. a—*p* < 0.001 cancer vs. urinary bladder control; b—*p* < 0.001 high-grade vs. low-grade urinary bladder cancer.

Figure 5 shows the actual activity of MMP-3 and MMP-10 in control and cancer tissues. The activity equals 263 pikokatals per kg of protein for MMP-3 and 140 pikokatals per kg of total protein for MMP-10 in normal human urinary bladder. Low-grade urinary bladder cancer tissues were characterized by an increase of actual activity of MMP-3. With the increase in grade of urinary bladder cancer, the measured activity decreased. The determined differences between both grades of cancer and the control tissue were significant (*p* < 0.001). In low-grade urinary bladder cancer, the actual activity of MMP-10 was more than four times higher. An increase in the grade of cancer resulted in a drastic decrease in actual activity of stromelysin-1, by about 24 times. The determined differences in actual activity of MMP-10 between both cancer grades and control tissue were significant (*p* < 0.001).

(5) Specific activity of MMP-3 and MMP-10

**Figure 6:**
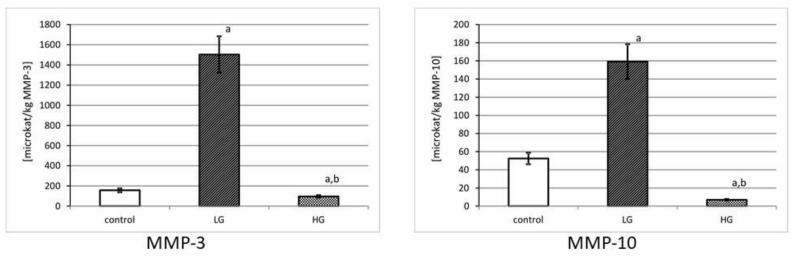
MMP-3 and MMP-10 specific activity in control tissue and low-grade (LG) and high-grade (HG) urinary bladder cancer. a—*p* < 0.001 cancer vs. urinary bladder control; b—*p* < 0.001 high-grade vs. low-grade urinary bladder cancer.

Figure 6 shows specific activity of MMP-3 and MMP-10. The highest specific activity of MMP-3 was found in low-grade urinary bladder cancer. The calculated specific activity of MMP-3 diminished with the increase in grade of urinary bladder cancer. The determined differences between both cancer grades and control tissue were statistically significant (*p* < 0.001). The control tissue revealed a middle value of that MMP-10 activity. The tissue of low-grade of urinary bladder cancer was characterized by a significant increase in specific activity of MMP-10 (*p* < 0.001). The high-grade cancer decreased that activity by almost 23 times (*p* < 0.001).

## 4. Discussion

Urinary bladder constitutes a significant organ in the human body. It may be structurally and functionally damaged as a result of an inflammation ending with a cancer. The fatality rate of urinary bladder cancer is very high, which is why this disease was investigated by many researchers [32].

The extracellular matrix of the bladder has different functions, not only as a structural scaffold or cells. It interacts with cells and influences them, whereas the role of structural proteins of the wall is to maintain the integrity of the impermeable bladder surface [1,2,16]. This dynamic structure plays a key role in cell migration, adhesion, tissue repair, and angiogenesis as well as in pathological processes including the modulation of cancer cell invasion [15].

Our study revealed that in urinary bladder cancer, there were great differences in the content of investigated metalloproteinases associated with the stage of cancer progression. It is well known that more than 57% of the dry weight of entire insoluble protein is constituted by collagen, the main extracellular matrix protein of a healthy urinary bladder. Such a huge collagen amount is related to its extensibility function during the bladder lumen filling [1]. The presence and the activity of matrix metalloproteinases are the factors affecting the degradation of collagen.

Primary, in order to assessment of relevant relation between cells quantity and extracellular matrix content, DNA content was estimated. We observed that DNA content was higher in both grades of urinary bladder cancer in comparison with healthy tissue. It may signify that ECM remodeling and activity of matrix metalloproteinases is far more intensified in cancer than in normal urinary bladder.

Secondly, we found a higher content of MMP-10 than of MMP-3 in healthy tissue. It shows that MMP-10 may be a more important enzyme for the extracellular matrix reconstruction in the urinary bladder. MMP-10 prevails over MMP-3 also in cancer tissues, but the MMP-10 content was similar in high-grade and low-grade tumors. Both low-grade and high-grade urinary bladder cancers contain a four-times lower level of MMP-3 than MMP-10. According to the above outcomes, it can suggest that the synthesis and the secretion of examined metalloproteinases out of cells were not inhibited.

The Western immunoblot test showed that the expression of both MMPs was similar for all investigated tissues except for MMP-10 in high-grade urinary bladder cancer. Bands with a molecular mass of about 48 kDa visible for both stromelysins may be a free active form of these enzymes [9,10,18,19]. Bands with a higher molecular mass demonstrated that the investigated metalloproteinases were present in complexes with other extracellular matrix proteins including TIMPs, or even the dimer formation [32]. The narrow band of 202 kDa visible for MMP-10 in the high-grade cancer may only be an enzyme in complexes as mentioned above. The extracellular matrix is a specific component of the tissue in which different proteins interact with one another, often without influencing the enzyme activity [1]

The measured actual activity, expressed in katals per kilogram of total protein content in tissue extract, allows comparing the investigated stromelysin activities. The actual activity of both stromelysins in normal bladder tissue is dissimilar, namely it is much higher for MMP-3 than for MMP-10. Since the metalloproteinases participate in the permanent process of remodeling of extracellular matrix, it seems that MMP-3 substantially contributes to the maintenance of ECM homeostasis in normal urinary bladder. The actual activity of stromelysin 2 in the low-grade urinary bladder cancer is higher in comparison to stromelysin 1, whereas it is much lower in high-grade urinary bladder cancer. This may be a further proof of varying roles of MMP-3s and MMP-10s in the extracellular matrix reconstruction process at different stages of the cancerous process.

The present study showed the activity of MMP-3 and MMP-10 in both low-grade and high-grade tumor cells. However, no correlation between the enzyme expression/activity and the cancer progression was found. No relationships between the tissue expression of MMP-3 and the neoplastic process progression and survival prognosis have been identified yet [9,10]. Additionally, the expression of MMP-10 does not indicate any relationship with the progression of bladder carcinoma [18].

It was demonstrated that MMP-3 was associated with the metastatic potential of various cancers [33,34]. No correlation between the MMP-3 tissue expression and the clinicopathological parameters or the disease prognosis was found. Moreover, based on circulating plasma MMP-3 levels it was not possible to distinguish between bladder cancer patients and healthy controls, and they were not predictive for the bladder cancer-specific survival [26,33]. Therefore, it can be concluded that MMP-3 has no diagnostic importance, and its prognostic utility in bladder cancer is disputable.

Based on the calculated specific activity, we found what part of the enzyme appeared in an active form with an active center without any inhibitor bound. For both MMPs, the specific activity decreased with increased histopatological tumor grade and gave inverse results. It was higher for MMP-3 than for MMP-10. It seems that the stromelysin 1 proteins are more enzymatically active than stromelysin 2 proteins.

The abovementioned differences between the results for both metalloproteinases can indicate their definite involvement in a particular period of growth as well as in differentiation of the cancer. The content of MMP-10 in all investigated tissues is much higher than of MMP-3. The actual and specific activity values of MMP-10 are presented in microkatals while the same parameters for MMP-3 are given in pikokatals. That is why MMP-10 seems to be much more important and valuable in the course of urinary bladder cancer than MMP-3. Considering the differences in all tissues, the catalytic ability of investigated enzymes rises in a low-grade urinary bladder cancer. Furthermore, the above outcomes indicate that cancerous cells of a high-grade urinary bladder tumor may silence the activity of MMPs. We can also suspect that much more molecules of MMP-10 were present in an active form compared to MMP-3. Based on the revealed differences in the activity of both stromelysins, their opposite participation in different phases of ECM remodeling and at different stages of tumor development was found. The control material originated from the same bladder, and we cannot exclude a carcinogenetic influence on the metabolism of the whole urinary bladder. The initiation of extracellular matrix components degradation may be assumed to be the most important stage in the tumor growth process. The tissue material was used for the study instead of urine or blood serum of the same patient because it gives a better reflection of the function of stromelysins in the tissue metabolism.

Summarizing, we noted different changes in the content of extracellular proteins in healthy and cancerous human urinary bladder. Compared to MMP-3, MMP-10 had a higher content, but a lower activity in all investigated tissues. Such a phenomenon indicates that there is difference on the regulation of activation and expression with reference to the investigated matrix metalloproteinases.

## Data Availability

The data that support the findings will be available on request under the corresponding author’s e-mail: jacek.kudelski@umb.edu.pl.
